# Overexpression of Phosphoribosyl Pyrophosphate Synthase Enhances Resistance of *Chlamydomonas* to Ionizing Radiation

**DOI:** 10.3389/fpls.2021.719846

**Published:** 2021-08-26

**Authors:** Sera Jung, Kwang Min Koo, Jaihyunk Ryu, Inwoo Baek, Soon-Jae Kwon, Jin-Baek Kim, Joon-Woo Ahn

**Affiliations:** ^1^Advanced Radiation Technology Institute, Korea Atomic Energy Research Institute, Jeongeup-si, South Korea; ^2^Advanced Process Technology and Fermentation Research Group, Research and Development Division, World Institute of Kimchi, Jeongeup-si, South Korea

**Keywords:** phosphoribosyl diphosphate synthase (PRPS), ionizing radiation, transcriptome analysis, DNA damage response (DDR), DNA repair

## Abstract

The enzyme phosphoribosyl pyrophosphate synthase (PRPS) catalyzes the conversion of ribose 5-phosphate into phosphoribosyl diphosphate; the latter is a precursor of purine and pyrimidine nucleotides. Here, we investigated the function of PRPS from the single-celled green alga *Chlamydomonas reinhardtii* in its response to DNA damage from gamma radiation or the alkylating agent LiCl. *CrPRPS* transcripts were upregulated in cells treated with these agents. We generated *CrPRPS*-overexpressing transgenic lines to study the function of CrPRPS. When grown in culture with LiCl or exposed to gamma radiation, the transgenic cells grew faster and had a greater survival rate than wild-type cells. *CrPRPS* overexpression enhanced expression of genes associated with DNA damage response, namely *RAD51*, *RAD1*, and *LIG1.* We observed, from transcriptome analysis, upregulation of genes that code for key enzymes in purine metabolism, namely *ribonucleoside-diphosphate pyrophosphokinase subunit M1*, *adenylate kinase*, and *nucleoside-diphosphate kinase*. We conclude that *CrPRPS* may affect DNA repair process via regulation of *de novo* nucleotide synthesis.

## Introduction

The enzyme phosphoribosyl pyrophosphate synthase (PRPS, EC 2.7.6.1) is an enzyme in the biosynthetic pathways of both purine and pyrimidine nucleotides, whether they are synthesized *de novo* or from salvage pathways. The enzyme catalyzes the conversion of ribose 5-phosphate to phosphoribosyl diphosphate (PRPP), a precursor of both purines and pyrimidines. The enzyme is a key intermediate for cellular metabolism of both carbon and nitrogen in living organisms (Hove-Jensen et al., 2016). Three classes of PRPS proteins have been identified based on their biochemical characteristics. Class I PRPSs, present in all living organisms, require Mg^2+^ and phosphate to function and are allosterically inhibited by adenosine diphosphate (ADP) (Krath and Hove-Jensen, 2001). Class II PRPSs, present in plants, are neither allosterically inhibited by ADP nor does their function dependent on phosphate (Krath and Hove-Jensen, 1999; Krath and Hove-Jensen, 2001). Both Class I and II PRPSs have been observed in *Arabidopsis.* A third class of PRPS, Class III, was detected in the archaeon *Methanocaldococcus jannaschii.* The activity of Class III enzymes is dependent on phosphate but is not allosterically inhibited by ADP (Kadziola et al., 2005). Approximately 80% of the metabolic flux through PRPP flux is directed to purine and pyrimidine metabolism (Hove-Jensen, 1988). In cells, nucleotides act primarily as subunits of nucleic acids or as energy carriers [particularly adenosine triphosphate (ATP) and guanine triphosphate (GTP)]; they also serve as precursors to nucleotide cofactors (Moffatt and Ashihara, 2002). Nucleotide metabolism and the cell cycle are related in that purines provide the energy and cofactors for the cell cycle; they also sustain DNA fidelity during replication (Ben-Sahra et al., 2013).

Phosphoribosyl pyrophosphate synthase (PRPS) has diverse functions in plants and animals, as described in a number of studies that we mention below. When *de novo* synthesis of nucleotides was reduced in potato and tobacco plants, their growth was reduced (Schröder et al., 2005). Similarly, enhanced growth and biomass accumulation were observed in *Arabidopsis* and *Nicotiana* transgenic plants that overexpressed PRPS genes from the fungus *Ashbya gossypii* (Koslowsky et al., 2008). In humans, abnormal expression of PRPS has been implicated in many diseases, including cancer (Ben-Sahra et al., 2013; Jing et al., 2019; Li et al., 2019). Suppression of PRPS expression in human neuroblastoma cells disrupted DNA synthesis and inhibited both neuroblastoma cell proliferation and tumor growth (Li et al., 2019). Mutation of PRPS1 in lymphoblastic leukemia cells increased their sensitivity to 5-fluorouracil, an inhibitor of nucleotide synthesis (Wang et al., 2018). Human colorectal cancer cells had greater expression of PRPS1 than normal cells and defective PRPS1 enzymatic activity caused arrest of the cell cycle and delayed cell proliferation in the cancer cells (Jing et al., 2019).

DNA damage can have various causes, including radiation and alkylating agents. Gamma radiation can damage DNA both directly and indirectly, the latter through the formation of free radicals. Lesions in the DNA associated with gamma radiation include single- and double-strand breaks (SSBs and DSBs, respectively), oxidized bases, and abasic sites (Cadet et al., 1999; Annex, 2000). Breaks in DNA are common. Mechanisms to repair SSBs include base excision repair, mismatch repair, and nucleotide excision repair. Repair of DSBs can be accomplished by one of two major pathways: homologous recombination or non-homologous end joining.

In this study, we investigated the function of PRPS from the single-celled green alga *Chlamydomonas reinhardtii*. Specifically, we were interested in its role in the cell’s response to DNA damage (DNA damage response, DDR). Expression of *CrPRPS* was assessed after DNA damage from gamma radiation or an alkylating agent, LiCl, and the survival rate of the algal cells was measured. We generated *CrPRPS*-overexpressing lines and compared them to wild-type (WT) algae to examine CrPRPS function. In addition, we performed transcriptome analysis to identify the molecular function of CrPRPS in purine metabolism and DDR. We believe this study is the first characterization of CrPRPS role in the DDR of *C*. *reinhardtii*.

## Materials and Methods

### Biological Material and Growth Conditions

We used the single-celled green alga *C*. *reinhardtii* strain cc125 in this study. Cells (1 × 10^5^ in 50 mL) were incubated in tris-acetate-phosphate (TAP) medium and then cultured at 25°C with 150 rpm shaking under constant white light (40 μmol photons m^–2^ s^–1^), unless noted otherwise. For NaCl treatment, TAP media containing 100, 200, and 300 mM NaCl were used. To identify effect of LiCl on *CrPRPS* expression in *C*. *reinhardtii*, 10, 20, and 30 mM of LiCl were utilized in TAP media. Three-day-old *C*. *reinhardtii* cells grown in TAP media were transferred to each TAP media containing different concentration of NaCl or LiCl. Samples were cultured for 24 h. For nitrogen-deplete condition, TAP without nitrogen (TAP-N; in which NH_4_Cl in TAP was replaced with KCl) were used. Three-day-old cells grown in TAP media was transferred to TAP-N media and then cultured for 2 days.

### Gamma Irradiation

Wild-type and *CrPRPS*-overexpressing cells were cultured for 3 days. Samples were irradiated with a gamma irradiator (^60^Co, approximately 150 TBq; Atomic Energy of Canada, Ltd., Ottawa, Ontario) for 2 h at the Korea Atomic Energy Research Institute. Samples were irradiated with either 80 or 200 Gy, which enabled us to assess dosage effects. Samples were harvested at 30 min after gamma irradiation.

### RNA Isolation and Quantitative Reverse Transcription (RT)-PCR

For RNA isolation, *C*. *reinhardtii* cells were harvested by centrifugation at 8,000 rpm for 5 min, resuspended in 1 mL Trizol reagent (Invitrogen, CA, United States), mixed for 10 min by vortexing, and incubated at room temperature for 5 min before centrifuging again. The supernatant was mixed with 250 μL chloroform (Sigma-Aldrich, MO, United States) by vortexing for 2 min, combined with an equal volume of phenol-chloroform-isoamyl alcohol (25:24:1 v/v; Sigma-Aldrich, MO, United States), mixed for 2 min by vortexing, and finally mixed with an equal volume of isopropanol and incubated for 1 h at 4°C. The RNA pellet was collected by centrifugation and washed with 1 mL 70% ethanol.

For RT-PCR, cDNA synthesis was performed using SuperScript III First-Strand Synthesis SuperMix (Invitrogen, CA, United States) according to manufacturer’s instructions. Quantitative RT-PCR was carried out with SYBR Premix EX Taq II (TaKaRa, Kyoto, Japan) using CFX Real-Time System (Bio-Rad, CA, United States). Conditions for quantitative RT-PCR analysis were as follows: 40 cycles at 92°C for 20 s, 55–60°C for 20 s, and 72°C 20 s. Primer sequences were as follows: *TubA*, 5′-CTC GCT TCG CTT TGA CGG TG-3′ and 3′-CGT GGT ACG CCT TCT CGG C-5′; *CrPRPS*, 5′-CTA TTT TAC ACG CCA GAC ACC-3′ and 3′-ACA AAG AGA TCA GGA AAG CC-5′; *RPA70a 2*, 5′-GCA CGA CTT CAA CGG CAG-3′ and 3′-GGT CAG GGA CTG CTT GGC-5′; *RAD1*, 5′-GGT GGA GCT GGT GAT GGT G-3′ and 3′-CTT GCA GTG GCG GTA CTT GT-5′; *RAD51a*, 5′-GCC TGG TTG TGG ACA G-3′ and 3′-GTT GGC CAC CTG ATT G-5′; *LIG1*, 5′-CGA CAC GTT CGA TGT GGT G-3′ and 3′-GAG CTG CTC GCT GAA G-5′; *Ku70*, 5′-CAG GTG TCG GTG TGT TCG AC-3′ and 3′-TCG CTC TCC CAC AGC TCC-5′. *AK3*, 5′-ACC CTG AAG GTC ATG ATT GC-3′ and 5′-ATC TCC ACG ACC ACC TCA TC-3′; *AK4*, 5′-GCC AAG AAG CTG GAT GAG AT-3′ and 5′-GGG CGA ACT TAA CGT GGT AG-3′; *FHIT*, 5′- AGG AGG TGT CAG ACC TGT GG-3′ and 5′-TCG TCA TTC TTG GGG AAG TC-3′; *GUCY1B*, 5′-GGC AAC ATGA CG ACC TAC CT-3′ and 5′-CCG ATG TTC TCA ACC GAC TT-3′; *NDK1*, 5′- CAC CGA GCA GAG CTA CAT CA-3′ and 5′-GGA CAG GTC CTC GTA GTG CT-3′; *NDK2*, 5′-CTA CAA GGA TCT GGC CTC CA-3′ and 5′-AGAGCCGTGGATCACGTTAC-3′; *RRM1*, 5′-AAC GAG TGC TTT GAG CCC TA-3′ and 5′-GAT CTC CCA CAC CGT CTT GT-3′. *TubA* was used as an internal control for quantification. Quantification was carried out using Bio-Rad CFX manager 3.1 program (Bio-Rad, CA, United States).

### Vector Construction and Generation of *CrPRPS*-Overexpressing Transgenic Lines

The DNA sequence for *CrPRPS* was obtained from Phytozom^1^ and specific primers of *CrPRPS* were designed for cloning. These contained the restriction enzyme sites *Hin*dIII and *Eco*RV. DNA fragments corresponding to *CrPRPS* cDNA were amplified using PCR. For generation of an overexpression construct, *CrPRPS* cDNA was inserted into the pCr102 vector using the restriction enzymes. For transformation of the *CrPRPS*-overexpressing construct into *C*. *reinhardtii*, cells in the mid-log phase (3.0 × 10^6^ cells mL^–1^) that had been cultured in TAP media for 3 days were collected by centrifugation at 3,000 rpm for 5 min. Cells were resuspended in TAP medium containing 60 mM sucrose. Gene Pulser Cuvette (Bio-Rad, CA, United States) and plasmid DNA (1 μg) were prepared for electroporation. Cell samples (250 μL) were mixed with plasmid DNA (1 μg) in a cuvette and incubated for 5 min at 16°C. Electroporation was conducted at 750 volts, 25 uF, and 200 Ω resistance. After electroporation, the cells in the cuvette were incubated for 10 min at room temperature. Transformed cells were transferred to TAP media containing 60 mM sucrose to allow them to recover, then they were incubated for 24 h with shaking at 120 rpm under white light. Transgenic colonies were selected on TAP media containing 50 μg mL^–1^ of hygromycin.

### Measurement of Cell Growth and Survival Rate

Cell growth rate was measured for the WT control and *CrPRPS*-overexpressing transgenic lines cultured with or without 30 mM LiCl in the medium; LiCl served as an alkylating agent. Cell density was used to assess growth rate and was measured as the OD at 750 nm taken every 24 h after inoculation with a UV spectrophotometer (UV-1800; Shimadz, Kyoto, Japan). Cell survival was assessed for irradiated cells and was determined by colony number. Approximately 300 cells were spread onto a solid TAP medium plate and colonies were counted at day 10 after spreading.

### Transcriptome Analysis

Two biological replicates of samples were prepared for transcriptome analysis. RNA isolation was performed as described above. Transcriptome analysis was performed as described by Koo et al. (2017b). Briefly, mRNA-Seq paired-end libraries were constructed using the Illumina TruSeq RNA Sample Preparation Kit v2 (Illumina, San Diego, CA, United States), and the KAPA library quantification kit (Kapa Biosystems, Wilmington, MA, United States) was utilized for quantification of the library according to the manufacturer’s instruction. The cDNA libraries were sequenced using an Illumina HiSeq2000 (Illumina). For short-read mapping, reads were mapped to reference transcripts using the bowtie software (Langmead et al., 2009).

### Statistical Analyses

One-way analyses (ANOVA) were carried out for statistical analyses of quantitative RT-PCR and plant growth measurement using R program (version 3.6.1). For transcriptome analysis, DEseq program was used.

## Results

### Expression of *CrPRPS* in *C*. *reinhardtii* in Response to Stress

**FIGURE 1:**
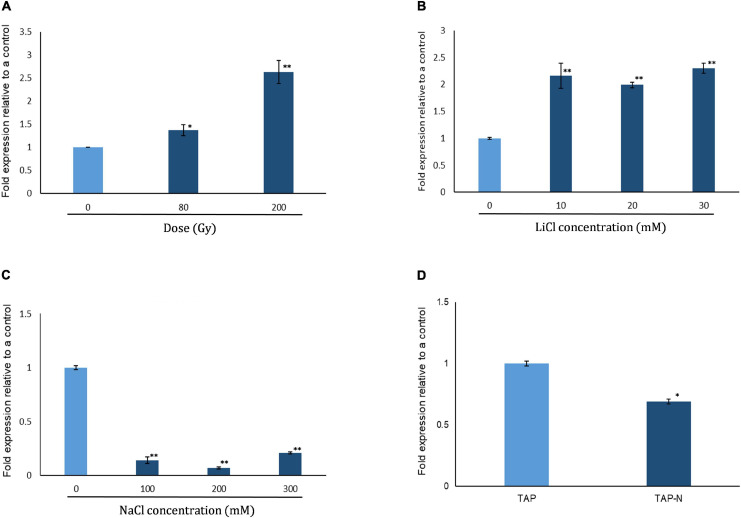
Expression of phosphoribosyl pyrophosphate synthase in *C*. *reinhardtii* strain cc125 (*CrPRPS*) as determined with quantitative RT-PCR. Cells grown in TAP media for 3 days were used. Cells were either subjected to radiation or stress conditions were imposed in culture. Cells exposed to: **(A)** Gamma radiation, **(B)** LiCl, **(C)** NaCl, and **(D)** nitrogen depletion. (Data represent the mean of three replicates ± SD; statistical analysis was carried out by one-way ANOVA; **, *p* < 0.01; *, 0.01 < *p* < 0.05).

We analyzed expression of *CrPRPS* in WT *C*. *reinhardtii* with quantitative RT-PCR in response to several types of stress, specifically gamma irradiation, nitrogen depletion, and treatment with NaCl and LiCl. As determined by the transcript levels relative to untreated algal cells, *CrPRPS* was induced after exposure to either 80 or 200 Gy gamma radiation (Figure 1A). Transcript levels were increased approximately 2.6- and 1.4-fold over the control when cells were exposed to 200 and 80 Gy gamma radiation, respectively. Treatment of the cells with LiCl also increased the transcript level of *CrPRPS* (Figure 1B) compared to the control cells, by more than 2-fold after exposure to all concentrations of LiCl (10–30 mM). In contrast, when we treated the cells with NaCl, we observed more than a 4-fold decrease in the transcript level of *CrPRPS* at all concentrations tested (Figure 1C) compared to the untreated cells. The level of *CrPRPS* transcripts was also slightly less in nitrogen-depleted cells than in cells grown in normal nitrogen levels (Figure 1D).

### Generation of *PRPS*-Overexpressing Transgenic Lines

**FIGURE 2:**
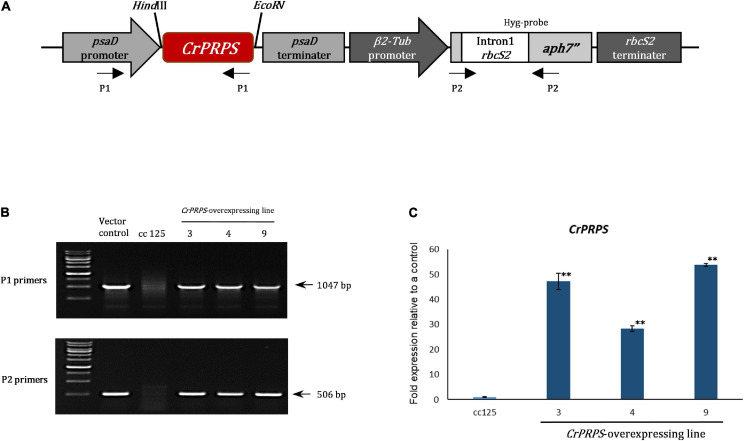
Generation of transgenic lines of *C*. *reinhardtii* that overexpressed phosphoribosyl pyrophosphate synthase (*CrPRPS*) and confirmation of enhanced expression. **(A)** The schematic diagram of the vector construct for *CrPRPS* overexpression. **(B)** Determination of the vector construct in genomic DNA of *CrPRPS*-overexpressing lines by PCR. **(C)** Comparison of transcript levels of *CrPRPS* between *CrPRPS*-overexpressing lines and the wild type, strain cc125. (Data represent the mean of three replicates ± SD; statistical analysis was carried out by one-way ANOVA; **p < 0.01).

We generated transgenic lines of *C*. *reinhardtii* that overexpressed *CrPRPS* compared to the WT strain cc125. Figure 2A shows the structure of the *CrPRPS*-overexpressing construct in which *CrPRPS* is controlled by the *psaD* promoter and terminator. We used genomic PCR to confirm the insertion of the *CrPRPS*-overexpressing construct into the genome of the transgenic lines (Figure 2B) and selected three transgenic lines that had the correct PCR products amplified by P1 and P2 primers. These were the independent transgenic lines designated line 3, 4, and 9; all had considerable overexpression of *CrPRPS*: approximately 45-, 24-, and 53-fold, compared to the WT, respectively (Figure 2C).

### *CrPRPS* Overexpression in Transgenic Lines of *C*. *reinhardtii* Confers Resistance to LiCl and Gamma Irradiation

**FIGURE 3:**
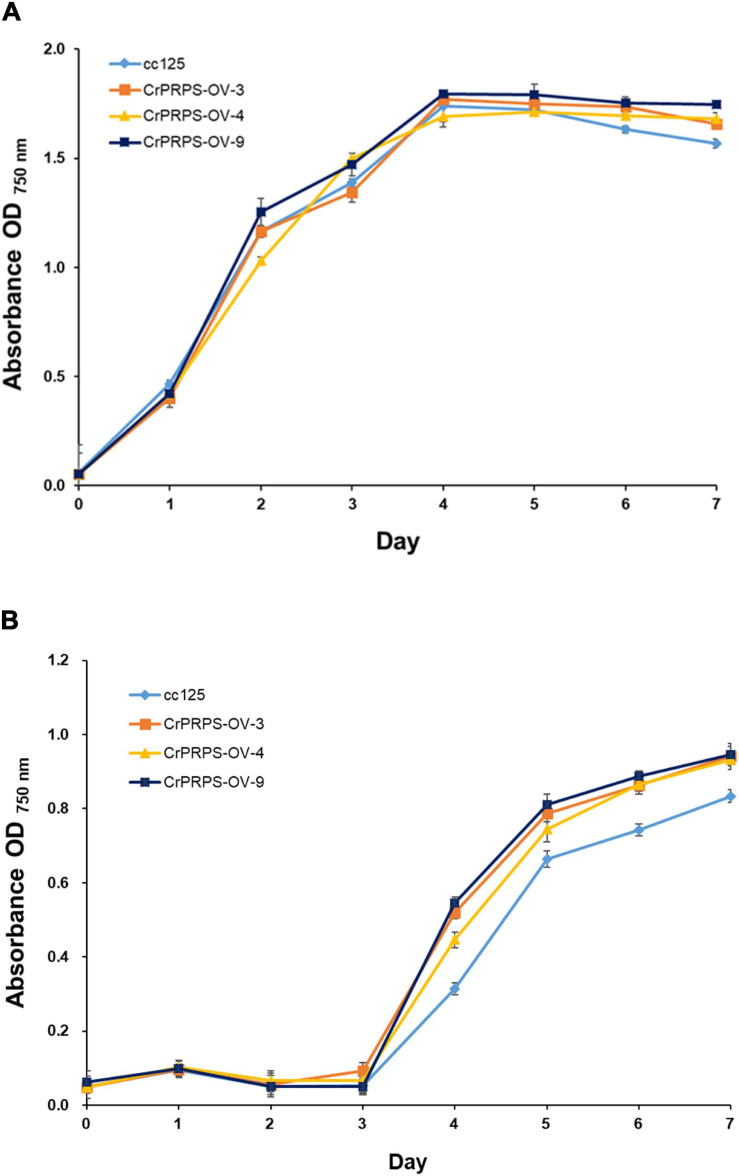
Growth of *C*. *reinhardtii* cells, either wild type (strain cc125) or from one of three independent transgenic lines (lines 3, 4, and 9) that overexpressed phosphoribosyl pyrophosphate synthase (*CrPRPS*), as measured by OD. Cells were exposed to: **(A)** No stress (media only) or **(B)** 30 mM LiCl in the media. (Data represent the mean of three replicates ± SD; statistical analysis was carried out by one-way ANOVA; *p* < 0.05).

We measured the growth of WT cells and cells of the transgenic lines with and without various stresses. There were no significant differences in growth between WT and cells from the transgenic lines in the absence of stress (grown on standard conditions in TAP media) (Figure 3A). However, cells of the *CrPRPS*-overexpressing lines grew faster than the WT cells when the media contained 30 mM LiCl (Figure 3B). In contrast, there was no significant difference in the growth of WT and transgenic cells when the media were depleted of nitrogen (data not shown).

**FIGURE 4:**
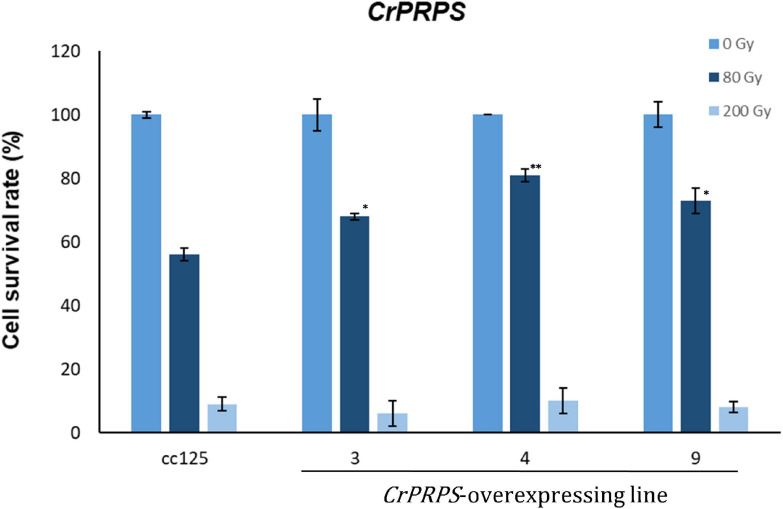
Survival rate of *C*. *reinhardtii* cells, either wild type (strain cc125) or from three independent transgenic lines (lines 11-3, 11-4, and 11-9) that overexpressed phosphoribosyl pyrophosphate synthase (*CrPRPS*), after exposure to different levels of gamma irradiation. For statistical analysis, transgenic lines were compared to cc125 with a same treatment. (Data represent the mean of three replicates ± SD; statistical analysis was carried out by one-way ANOVA; **p < 0.01; *0.01 < *p* < 0.05).

Survival rates of cells from the *CrPRPS*-overexpressing lines were measured after 80 and 200 Gy gamma irradiation. More of the cells from the transgenic lines survived the 80 Gy radiation treatment than the WT cells (Figure 4): lines 3, 4, and 9 had survival rates of 68, 81, and 73%, respectively, compared with the WT survival rate of 56%. When the dosage of gamma radiation was greater, 200 Gy, there was no significant differences in cell survival rates between the transgenic lines and the control.

### Overexpression of *CrPRPS*-Overexpression in *C*. *reinhardtii* Enhanced Expression of Genes Involved in DDR

**FIGURE 5:**
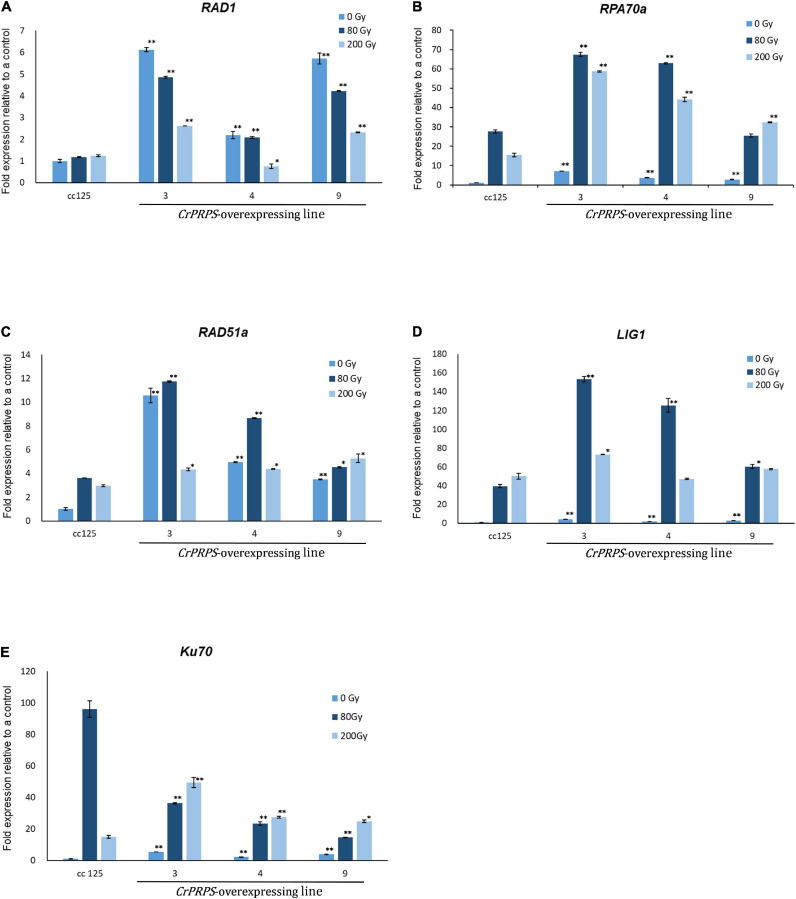
Expression profiles of genes involved in the DNA damage response in *C*. *reinhardtii* cells, either wild type (strain cc125) or from three independent transgenic lines (lines 3, 4, and 9) that overexpressed phosphoribosyl pyrophosphate synthase (*CrPRPS*), after exposure to different levels of gamma irradiation. The genes examined were: **(A)** *RAD1*, **(B)** *RPA70a*, **(C)** *RAD51a*, **(D)** *LIG1*, and **(E)** *Ku70*. (Data represent the mean of three replicates ± SD; statistical analysis was carried out by one-way ANOVA; **p < 0.01; *0.01 < *p* < 0.05).

We examined the molecular mechanism of *CrPRPS* on the cells’ response to DNA damage. To this end, the levels of expression of the genes associated with DDR were assessed. In normal, unstressed conditions, the expression levels of *RAD1* were approximately 2.2–6.1-fold greater in the *CrPRPS*-overexpressing lines than in the control (Figure 5A) and were also greater than in the control following either dose of gamma radiation.

The expression of replication protein A 70 kDa DNA-binding subunit (*RPA70a*) was upregulated from approximately 2.7- to 7-fold in the *CrPRPS* overexpressing lines compared to the WT under normal conditions without gamma irradiation (Figure 5B). Expression was greater in all three lines following gamma irradiation than in the WT. Similarly, we detected transcriptional induction of *RAD51a* in the *CrPRPS* overexpressing lines compared to the WT control, with and without gamma irradiation (Figure 5C). In addition, transcript levels of both *DNA ligase 1* (*Lig1*) and *Ku70* were greater in the *CrPRPS*-overexpressing lines than in the WT under both gamma-irradiated and non-irradiated conditions with the exception of *Ku70* expression at 80 Gy (Figures 5D,E).

### Transcriptome Analysis for Purine Metabolism in *CrPRPS*-Overexpressing Transgenic Lines

**FIGURE 6:**
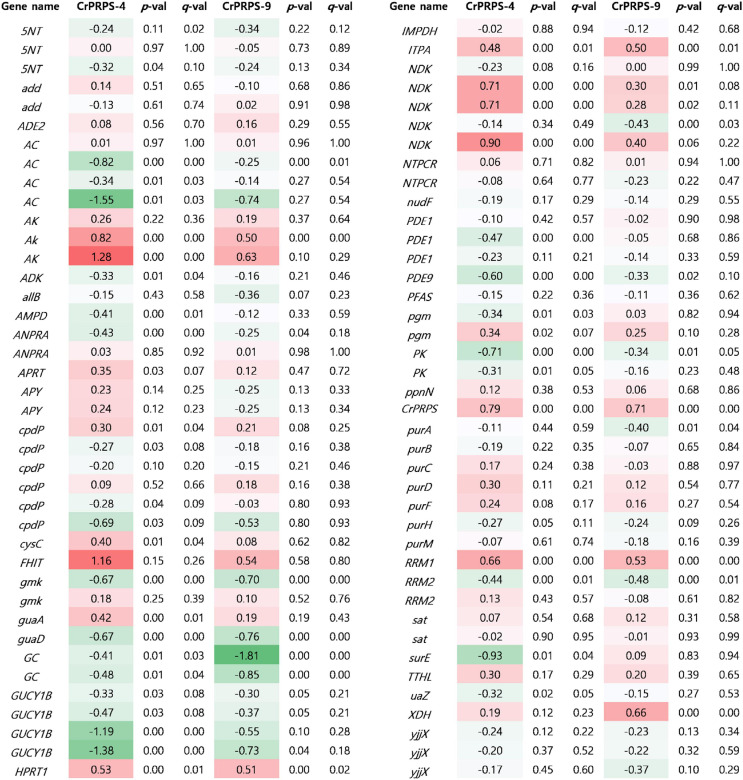
Comparative transcriptome analysis for genes associated with purine metabolism in *C*. *reinhardtii* cells, either wild type (strain cc125) or from transgenic lines that overexpressed phosphoribosyl pyrophosphate synthase (*CrPRPS*). Values indicate changes (log_2_). Statistical analysis was performed using DEseq program. Gene information was listed in Supplementary Table 2. *5NT*; *5*′*-nucleotidase, add; adenosine deaminase*, *ADE2*; *phosphoribosylaminoimidazole carboxylase*, *AC*; *adenylate cyclase*, *AK*; *adenylate kinase*, *ADK*; *adenosine kinase*, *allB*; *allantoinase*, *AMPD*; *AMP deaminase*, *ANPRA*; *atrial natriuretic peptide receptor A*, *APRT*; *adenine phosphoribosyltransferase*, *APY*; *apyrase, cpdP*; *3*′*,5*′*-cyclic-nucleotide phosphodiesterase*, *cysC*; *adenylylsulfate kinase*, *FHIT*; *bis(5*′*-adenosyl)-triphosphatase*, *gmk*; *guanylate kinase*, *guaA*; *GMP synthase*, *guaD*; *guanine deaminase*, *GC*; *guanylate cyclase*, *GUCY1B*; *guanylate cyclase soluble subunit beta*, *hprT*; *hypoxanthine phosphoribosyltransferase*, *IMPDH*; *IMP dehydrogenase*, *ITPA*; *inosine triphosphate pyrophosphatase*, *ndk*; *nucleoside-diphosphate kinase*, *NTPCR*; *nucleoside-triphosphatase*, *PDE1*; *calcium-dependent 3*′*,5*′*-cyclic nucleotide phosphodiesterase*, *PDE9*; *high affinity cGMP-specific 3*′*,5*′*-cyclic phosphodiesterase 9*, *PFAS*; *phosphoribosylformylglycinamidine synthase*, *pgm*; *phosphoglucomutase*, *PK*; *pyruvate kinase, ppnN*; *pyrimidine/purine-5*′*-nucleotide nucleosidase*, *purA*; *adenylosuccinate synthase*, *purB*; *adenylosuccinate lyase*, *purC*; *phosphoribosylaminoimidazole- succinocarboxamide synthase*, *purD*; *phosphoribosylamine glycine ligase*, *purF*; *amidophosphoribosyltransferase*, *purH*; *phosphoribosylaminoimidazolecarboxamide formyltransferase*, *purM*; *phosphoribosylformylglycinamidine cyclo-ligase*, *RRM1*; *ribonucleoside-diphosphate reductase subunit M1*, *RRM2; ribonucleoside- diphosphate reductase subunit M2*, *sat*; *sulfate adenylyltransferase, surE; 5*′*-nucleotidase*, *TTHL*; *5-hydroxyisourate hydrolase*, *uaZ*; *urate oxidase*, *XDH*; *xanthine dehydrogenase/oxidase*, *yjjX*; *inosine/xanthosine triphosphatase*.

Expressional changes of genes involved in purine metabolism were determined in *CrPRPS*-overexpressing lines by transcriptome analysis (Figure 6). Three homologous genes of *nucleotide-diphosphate kinase* (*NDK*), homologous genes of *adenylate kinase* (*AK*), and *ribonucleoside-diphosphate pyrophosphokinase subunit M1* (*RRM1*) were all transcriptionally upregulated in the *CrPRPS*-overexpressing lines compared to the WT control (Figure 6). In the transcriptome analysis, we also observed the induction of *CrPRPS* expression in cells of the transgenic lines (Figure 6). This confirms our earlier result (Figure 2C), in which we detected higher transcript levels for *CrPRPS* in the transgenic lines than in the control cells.

Based on the transcriptome study, we also observed fewer *guanylate cyclase soluble subunit beta* (*GUCY1B*) and *adenylate cyclase* (*AC*) transcripts in the *CrPRPS*-overexpressing lines than in the WT (Figure 6). Annotated transcripts from the transgenic lines by transcriptome analysis are shown in Supplementary Table 1.

### Confirmation of Gene Expression on Purine Metabolism in *CrPRPS*-Overexpressing Transgenic Lines

**FIGURE 7:**
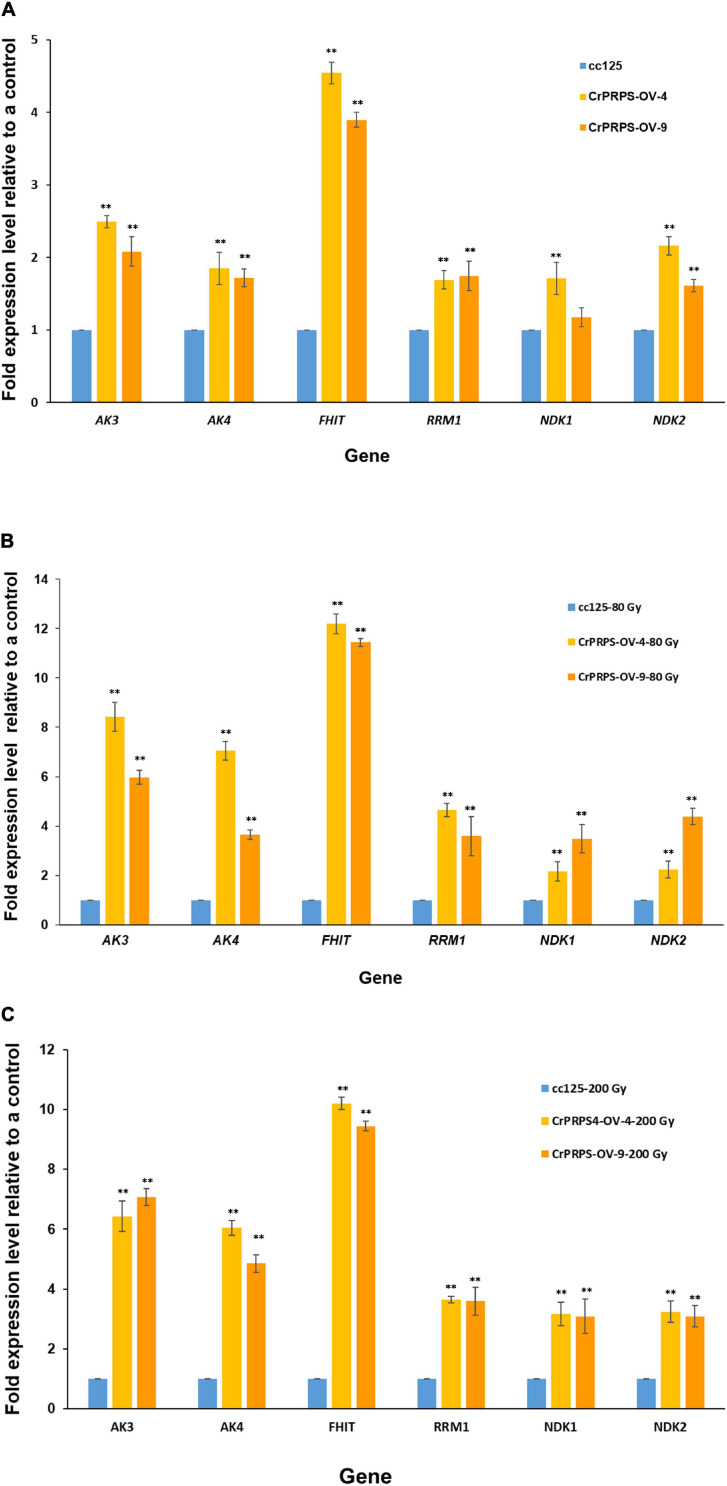
Expression of genes involved in purine metabolism in *CrPRPS-*overexpressing transgenic lines (lines 4 and 9) before and after gamma irradiation. Expression levels were determined by quantitative RT-PCR: **(A)** non-irradiation, **(B)** 80 Gy gamma irradiation, **(C)** 200 Gy gamma irradiation. *AK3*; Cre12.g494850.t1.2, *AK4*; Cre12.g557600.t1.1, *FHIT*; Cre17.g698266.t1.1, *RRM1*; Cre12.g492950.t1.2, *NDK1*; Cre16.g650550.t1.2, *NDK2*; Cre07.g325734.t1.1. (Data represent the mean of three replicates ± SD; statistical analysis was carried out by one-way ANOVA; **p < 0.01).

Differential expression of genes such as *AKs*, *NDKs*, and *RRM1* on purine metabolism were identified by the transcriptome analysis (Figure 6). Expression levels of these genes were determined in *CrPRPS*-overexpressing transgenic lines before and after gamma irradiation by quantitative RT-PCR. Under non-irradiation condition, transcript levels of *AK3* were induced over 2-fold in *CrPRPS*-overexpressing transgenic lines (Figure 7A). *AK4* and *RRM1* showed over 1.8-fold induction. In addition, upregulation of *NDK2* transcript was detected by CrPRPS overexpression (Figure 7A). *FHIT* was upregulated over 3.8-fold in both transgenic lines. After both 80 and 200 Gy gamma irradiation, expression levels of all genes were enhanced in comparison with the non-irradiated samples (Figures 7B,C).

## Discussion

Phosphoribosyl pyrophosphate synthase is an important enzyme in nucleotide synthesis in all living organisms. It has been implicated in many aspects of metabolism, including maintaining the integrity of DNA during replication and repair after damage. However, the specific function of PRPS on the DDR has not been identified. Here, we have chosen to study the role of this enzyme in DDR in the single-celled green alga *C. reinhardtii.* To aid this study, we generated transgenic algal lines that overexpressed *CrPRPS* (Figure 2).

We first determined that the transcript level of *CrPRPS* was increased by a DNA damaging agent, gamma radiation, and by a purported DNA damaging agent, LiCl (Figure 1). Gamma radiation causes DNA damage via direct and indirect action (Desouky et al., 2015). The molecular mechanism of LiCl action is not completely understood, but it is proposed to act as a DNA-damaging agent (Duffy et al., 2014; Stampone et al., 2020). From this result, we suggest that the DNA damage upregulates the transcription of *CrPRPS*.

Repair of DNA damage induced by radiation or genotoxic agents is affected by purine and pyrimidine precursors in human cells (Cleaver, 1973). Purine metabolism also regulates DNA repair in glioblastoma (Peifer et al., 2012). Given this background, we hypothesized that the *CrPRPS*-overexpressing cells would be more resistant to DNA damage by radiation than WT cells. We tested this by exposing the cells to gamma radiation and measuring the cell survival rate. The irradiated transgenic cells did, indeed, have enhanced survival to irradiation than the control cells (Figure 4). We suggest that *CrPRPS* overexpression in *C*. *reinhardtii* enhances purine metabolism and consequently enhances resistance to gamma radiation.

We examined the expression patterns of several DNA repair genes. The RAD9/RAD1/HUS1 complex is a DNA damage sensor that, together with the kinase ATR, responds to stalled replication forks (Griffith et al., 2002). Figure 5A shows that *CrPRPS*-overexpressing cells had greater *RAD1* expression than a WT control whether or not they received gamma irradiation. We also assessed the transcript levels of *RPA70A*, *RAD51*a, and *Ku70.* Like the expression of *RAD1*, these genes were all significantly induced in the *CrPRPS*-overexpressing transgenic lines before and after gamma irradiation (Figures 5B,C,E). They have at least partially described roles in DNA repair, as briefly described below. RPA, the single-strand binding protein, is involved in DNA repair and replication (Aklilu et al., 2014). In *Arabidopsis*, *RPA70* deletion increased sensitivity to replication stress from hydroxyurea, and its expression was induced by DNA damage (Takashi et al., 2009). Koo et al. (2017a) studied DNA damage repair genes in *C. reinhardtii.* When the algal cells were exposed to gamma irradiation, they observed that two paralogs of *RPA70s* were transcriptionally induced and that *RAD51a* and *Ku70* expression were enhanced. RAD51 is important in homologous recombination repair and the Ku70/Ku80 heterodimer binds to DNA double strand break ends for non-homologous end joining (NHEJ) repair. More generally, it has been noted that nucleotide metabolism and cell cycle progression are correlated. Purines are involved in the maintenance of DNA fidelity during replication (Ben-Sahra et al., 2013). Jing et al. (2019) reported cyclin-dependent kinase 1-dependent activation of PRPS1and loss of PRPS1 enzymatic activity arrested cell cycle progression and reduced cell proliferation. Based on this background and our results, it may be that *CrPRPS* overexpression modulates replication stress and DDR in *C*. *reinhardtii*.

Phosphoribosyl pyrophosphate synthase is a key enzyme in purine metabolism, including its biosynthesis. Purines have diverse cellular functions, including energy metabolism and signaling pathways (Cordell et al., 2008; Guimarães and Londesborough, 2008). From our transcriptome and quantitative RT-PCR data, we conclude that expression of *AKs* and *RRM1* were induced in *CrPRPS*-overexpressing lines (Figures 6, 7). AK is an essential enzyme for nucleotide synthesis in purine metabolism and required for reversible interconversion of ATP and AMP to two ADP (Tükenmez et al., 2016). During cell division, AK also supplies energy and metabolic signaling (Dzeja et al., 2011; Zhang et al., 2014). Ribonucleotide reductase is a heterotetramer, composed of two large RRM1 subunits and two small RRM2 subunits. Ribonucleotide reductase is an essential enzyme for catalyzing *de novo* synthesis of deoxyribonucleosides prior to DNA synthesis (Parker et al., 1995). Sagawa et al. (2017) reported that RRM1 knockdown led to induction of genes involved in both DDR and the p53 pathway. Furthermore, cell-cycle-dependent phosphorylation of RRM1 enhanced ribonucleotide reductase activity (Shu et al., 2020).

In the present study, we found transcriptional upregulations of *NDKs* in *CrPRPS*-overexpressing *C*. *reinhardtii* lines via transcriptome analysis and quantitative RT-PCR (Figures 6, 7). NDKs are well-conserved enzymes in all living organisms and catalyze the exchange of phosphate between di- and tri-phosphate nucleosides (Agarwal et al., 1978). A major role of NDKs is to maintain an adequate supply of triphosphates for DNA and RNA synthesis (Hama et al., 1991). NDKs are multifunctional proteins: human NDKs (NME-H1 and NME-H2) have DNA binding activities and may be transcription factors (Puts et al., 2018), and there are reports that that NME-H1 is related to repair of DNA damage induced by gamma radiation, UV, or bleomycin (Yoon et al., 2005; Jarrett et al., 2012; Radić et al., 2020). In addition, many studies have reported that NDKs interact with, and cleave, DNA, so they may be associated with DNA processing (Kumar et al., 2005; Hammargren et al., 2007; Miranda et al., 2008).

Interestingly, downregulations of *AC* transcripts were observed in *CrPRPS*-overexpressing lines (Figure 6). AC catalyzes the conversion of ATP to cyclic AMP (Zhang et al., 1997). cAMP signaling inhibited repair of gamma radiation-induced DNA damage via enhancing degradation of XRCC1 in lung cancer cells (Cho and Juhnn, 2012). Furthermore, induction of cAMP level activated cAMP-dependent protein kinase, which resulted in inhibition of DNA damage repair in fission yeast (Lee et al., 2001). Therefore, reduction of *AC* transcript indicates activation of DNA repair in *CrPRPS*-overexpressing *C*. *reinhardtii* lines.

If we consider our results from this study in the light of this evidence, we conclude that the transcriptional inductions of components in purine metabolism resulting from *CrPRPS* overexpression activates purine metabolism in *C*. *reinhardtii*, and that these downstream components of CrPRPS in purine metabolism are correlated with DDR and cell cycle regulation. We suggest that CrPRPS plays a key role in DDR during cell cycle progression.

## Conclusion

**FIGURE 8:**
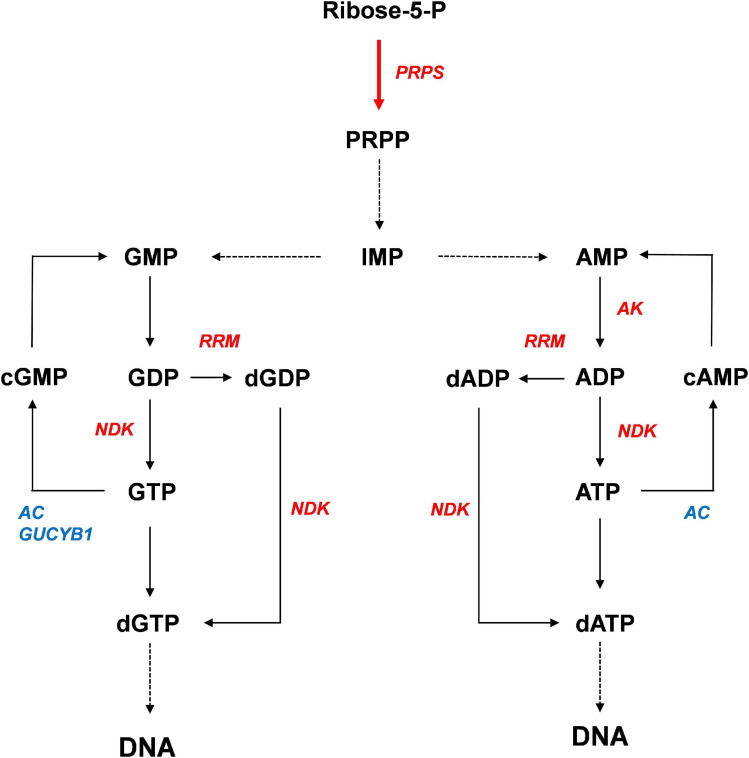
Proposed model of the enhancement of DNA repair by activation of purine metabolism. Red and blue colors indicate expressional up-regulation and down-regulation, respectively.

Based on our results, we propose a model for activation of purine metabolism by *CrPRPS* overexpression (Figure 8). According to our model, *CrPRPS* overexpression results in inductions of *RRM*, *NDK*, and *AK* expression, which enhances *de novo* nucleotide synthesis. This study supports the idea that *CrPRPS* may be a key regulator for DDR via regulation of purine metabolism in *C*. *reinhardtii*. This is the first report of the role of CrPRPS in DDR in the green alga *C*. *reinhardtii*.

## Data Availability Statement

The original contributions presented in the study are publicly available. This data can be found here: National Center for Biotechnology Information (NCBI) BioProject database under accession number PRJNA738545 (https://www.ncbi.nlm.nih.gov/bioproject/PRJNA738545).

## Author Contributions

J-WA wrote the manuscript, analyzed transcriptome data, and arranged all data. SJ generated transgenic lines. KMK and SJ carried out RT-PCR analysis and helped to design experiments. KMK performed transcriptome analysis. IB helped to analyze transcriptome data. J-BK and S-JK interpreted data. All authors contributed revision of the manuscript, read, and approved the manuscript.

## Conflict of Interest

The authors declare that the research was conducted in the absence of any commercial or financial relationships that could be construed as a potential conflict of interest.

## Publisher’s Note

All claims expressed in this article are solely those of the authors and do not necessarily represent those of their affiliated organizations, or those of the publisher, the editors and the reviewers. Any product that may be evaluated in this article, or claim that may be made by its manufacturer, is not guaranteed or endorsed by the publisher.

## Footnotes

1
https://phytozome.jgi.doe.gov/pz/portal.html#!info?alias=Org_Creinhardtii
